# Evaluation of the Retrospective LACE Index in Predicting the Risk of Readmission in Patients with Hereditary Angioedema in an Emergency Department

**DOI:** 10.1155/2023/8847030

**Published:** 2023-10-20

**Authors:** Meltem Songur Kodik, Ozlem Inci, Zeynep Dila Çetin, Emine Nihal Mete Gokmen, Funda Karbek Akarca

**Affiliations:** ^1^Ege University, Faculty of Medicine, Emergency Department, Izmir, Turkey; ^2^Ege University, Faculty of Medicine, Izmir, Turkey; ^3^Ege University, Faculty of Medicine, Division of Allergy and Immunology, Department of Internal Medicine, Izmir, Turkey

## Abstract

This study aimed to calculate the LACE index in patients who admitted to the emergency department (ED) with hereditary angioedema (HA) diagnosed and to predict recurrent admissions of patients. In this single-center study, patients aged 18 or higher who were admitted to the ED diagnosed with HA were included over a 12-year period. 35 patients diagnosed with code E88.0 were evaluated according to electronic file records. The number of admissions to the ED in the last 6 months was 2. The LACE index was 4, and risk was 71.4%. The patients admitted to the hospital in the last 30 days had a higher rate of admission to the hospital in the last 6 months (*p* < 0.001). The LACE index at admission predicted 30 days admission with (AUC = 0.75, 95% CI (0.56–0.91)) acceptable discrimination. The LACE index and the number of admissions in the last 6 months included in the evaluation can be considered predictive in recurrent ED admissions of HA patients. However, the distribution of LACE-risk groups is no priority. Therefore, the low-, medium-, or high-risk level of LACE index values should be not taken into consideration in readmission of such patients.

## 1. Introduction

Hereditary angioedema (HA) is a rare disease, usually autosomal dominant, characterized by recurrent but unpredictable urticarial skin and mucosal swellings. HA is mainly divided into two depending on the C1 inhibitor (C1INH) level and function. The most common type of C1 inhibitory function is called HA-C1INH due to C1 inhibitor deficiency, and the less common type of the C1 inhibitory level and normal function is C1 inhibitory normal HA-nC1INH. Both the C1 inhibitor level and C1 inhibitor function are low in approximately 85% of HA cases due to C1 inhibitor deficiency (type I HA-C1INH), and approximately, 15% have low C1 function but normal or high C1 inhibitor levels [1].

The most common localizations include the extremities, gastrointestinal tract, genital area, and upper respiratory tract [2].

The frequency and severity of HA attacks vary from person to person and even among family members carrying the same mutation. Attacks are self-limiting, and it may take up to 72–120 hours for symptoms to fully resolve [3]. Gastrointestinal system attacks cause very severe abdominal pain. Patients have been admitted to the emergency department (ED) an average of 5 times a year [4]. Airway edema in C1 inhibitor deficiency (C1INH-HA) has been reported to have a 15–34% mortality rate [5].

Emergency room doctors often encounter patients with angioedema. In the USA, the prevalence of angioedema and/or urticaria was found to be 25% in the population, and it was calculated that it causes more than 1,000,000 emergency admissions per year. Of these, approximately, 110,000/year were coded as angioedema (hereditary or acquired) and 979,400 as allergic reactions [6]. It has been reported that 2 out of every 100,000 emergency visits in the USA and 37 out of 10,000 in Italy are due to HA [7]. The true prevalence of HA remains unknown; however, the (probable) prevalence calculated by dividing the patients diagnosed with HA by the general population in some European countries has been reported as 1 : 10 000–1 : 50 000 [8]. In Turkey, it has been reported that a patient with HA can be diagnosed in an average of 20 years [9]. The most important reason for this is that it is the symptoms of the disease that are mixed with allergic angioedema, familial Mediterranean fever, and acute abdomen and that HA is not considered in differential diagnosis [10].

Angioedema in HA attacks does not respond to allergic angioedema treatment, therefore making a correct diagnosis is critical [11]. The mortality rate due to laryngeal edema in undiagnosed cases is approximately 30% [5].

Recent emergency service studies have shown that 9–59% of unplanned readmissions can be avoided if appropriate precautions are taken [12]. The LACE index, a cross-condition tool, predicts premature death or unplanned readmissions after hospital discharge [13] The National Early Warning Score (NEWS) can be used when patients are admitted to the hospital in an emergency and also in the prehospital period for the assessment of acute disease severity [14, 15].

Despite the availability of current guidelines for specific treatments in the treatment of HA attacks, HA morbidity and mortality rates remain important. HA attacks are an important socioeconomic burden as well as an important medical problem that requires special treatment. HA progresses with recurrent attacks of angioedema without urticaria and is generally treated by emergency physicians. However, there are no studies evaluating readmissions in this patient group.

The present study aimed to calculate the prediction of recurrent admission and mortality utilizing the LACE, NEWS, and NEWS2 scores, which are frequently used emergency service scores, in HA patients admitted to the ED.

## 2. Methods

### 2.1. Study Design

The study was planned as a single-center and retrospective study in Ege University Hospital Emergency Medicine Clinic. The research unit is a third-level reference health center in Izmir, Turkey, and approximately, 190,000 patients are cared for per year at the center. The research protocol was approved by the Ege University Clinical Research Ethics Committee (Date: 06.05.2021, no: 21-6.1T/67). All methods were carried out in accordance with relevant guidelines and regulations. Informed consent was obtained from all subjects and/or their legal guardian(s).

### 2.2. Settings

Patients aged 18 or higher who were admitted to the Ege University Emergency Service between 01.01.2009 and 01.06.2021 and were diagnosed with HA previously or at the time of admission were included in the study. A retrospective, single-center study was conducted to evaluate age, sex, clinical characteristics, and the LACE, NEWS, and NEWS2 scores in 35 patients who were diagnosed with HA previously or at the time of admission in the last 12 years.

### 2.3. Data Collection

Patient information included in the study was transmitted by the “electronic file and billing unit.” Based on diagnosis code E88.0, 60 patients were identified. Patients who did not meet the criteria due to lack of data were not included in the study. Data on demographics, clinical features, comorbidities, vital signs, and outcomes were reviewed from medical records. The data were used in the calculation of the LACE, NEWS, and NEWS2 scores.

### 2.4. Variables

The demographic data, age, gender, family history, presence of comorbidity, readmission in the last 6 months, HA type of the patients, treatment and duration of administration in the ED, symptoms on admission, angioedema localization and duration, and discharge and hospitalization status, in addition to the scoring models including LACE, NEWS, and NEWS2, comprised the main variables of the research:

- The LACE index, a cross-condition tool, predicts premature death or unplanned readmissions after hospital discharge [13]. The LACE index measures L: length of stay, A: acuity of admission, C: comorbidity of the patient measured with the Charlson comorbidity index [16], and E: emergency department use and admissions in the last 6 months.
- The National Early Warning Score (NEWS) is an important tool developed by the Royal Academy of Medicine that discriminates and responds to clinical worsening in adult patients, contributing to patient safety and patient improvement [14]. NEWS can be used when patients admitted to the hospital in an ED and also in the prehospital period for the assessment of acute disease severity [15].
- NEWS2 is an effective predictor of mortality for patients presenting to the ED. The results also suggested a NEWS2 value of 0-1 can identify a very low-risk group within the ED [17, 18].

### 2.5. Statistical Analysis

Statistical analyses were carried out using SPSS for Windows, version 26.0 (Chicago, IL, USA). Results were presented as the mean and standard deviation for numerical data, frequency, and percentage for categorical variables. Also, the statistical values of all tests are shown in the tables.

The conformity of the variables to normal distribution was evaluated utilizing the Kolmogorov–Smirnov test. The chi-square test or Fisher's exact test was utilized to compare categorical variables. The independent samples *t*-test was adopted to compare parametric variables. A logistic regression model was established for the prediction of multiple admissions to the ED and to identify the affecting independent variables. For statistical significance, a *p* value <0.05 was considered significant.

## 3. Results

In the study, a total of 35 patients aged 18 or higher diagnosed with HA were examined. Of these patients, 19 (54.3%) were female and 16 (45.7%) were male. The number of admissions to the ED in the last 6 months of all patients was calculated, excluding their last admission, as 1 (range 1–3). Among all the patients, only 1 patient was observed as intubated. The most common localization of angioedema was the gastrointestinal tract. Also, edema in the uvula was observed. Angioedema duration was calculated as 24 hours (range 1–168 hours). None of the patients had urticaria. Family history was present in 32 patients (91.4%). Trauma-related angioedema was detected in only 1 patient. As a treatment protocol, 1000 IU of the C1 inhibitor concentrate was administered to 24 patients (68.6%). 75% of type I HA cases received 1000 IU of the C1 inhibitor concentrate, and 3 of 6 patients with type II HA received 1000 IU of the C1 inhibitor concentrate. The C1 inhibitor concentrate was 1000 IU administered in 30 minutes (median value) (range 10–240 minutes) from the time the patient was admitted to the EU. In total, 4 patients were hospitalized (11.4%). Hospital stay was calculated as 4 days (median value) (range 1–17 days).

The LACE index, NEWS, and NEWS2 were calculated in all patients. The LACE index was calculated as 4 (range 3–11). Calculating the LACE index risk, 25 patients were found to have low risk (71.4%). The remaining 8 patients had moderate risk, and 2 patients had high risk.

Table 1 presents the baseline characteristics of all patients in the study.

A total of 7 of all patients were readmitted to the hospital within 30 days. Patients who were readmitted to the hospital in the last 6 months were evaluated in the LACE index. (Table 2). The LACE-risk groups were divided into three: the low-risk group (0–4 points), the medium-risk group (5–9 points), and the high-risk group (>9 points); the 30-day emergency admission distribution among these groups was statistically not significant (*p* = 0.069).

The LACE index at admission predicted 30 days admission with (AUC = 0.75, 95% CI (0.56–0.91)) acceptable discrimination. In the present study, the LACE index of patients with recurrent admissions in the last 6 months was calculated as 4.2, and the LACE index of patients with a single admission was calculated as 4.7. Statistically, the LACE index was found to be significant in the 30-day readmission (*p* = 0.030).

## 4. Discussion

HA is difficult to diagnose due to its rarity and its symptoms being confused with allergy, acute abdomen, or FMF clinic. Particularly, edema of the upper airway tract can result in death in cases that are not properly diagnosed and treated.

In the present study, 12-year data (2009–2021) were examined and 35 patients were included in the study. Of the patients, 54.3% were female, and the mean age was 37 (range: 18–71 years).

Also, 91.4% of the patients with autosomal inherited HA [1] had a family history. Of the cases, HA type 1 was the most common (80%).

In studies on this subject, the duration of HA attacks, angioedema localization, and symptoms have been reported to differ from person to person [2, 4]. In the present study, the duration of angioedema was calculated as 24 hours and the most common angioedema localization was the gastrointestinal system (37.2%). The most common complaint of admission to the ED was abdominal pain. Angioedema secondary to trauma was detected in only one patient.

The most important differential diagnosis that distinguishes HA from allergic angioedema is the possible presence of urticaria [1]. None of the patients in the present study had urticaria.

In studies in the literature, it was calculated that patients diagnosed with HA were admitted to the ED an average of 5 times a year [4]. The median number of admissions to the ED in 6 months was calculated as 1 [1, 3], excluding the last admission. In total, the number of patients admitted to the ED in the last 6 months was 2. The low rates of admission in the present study may be due to the fact that the hospital in which the study was conducted is a last-line hospital, the delayed diagnosis and treatment of HA patients, long waiting times in the ED, and the possible fact that patients were admitted to other hospitals since the study hospital is a third-level hospital.

HA patients have a mortality rate of 15–34% due to airway edema during an attack [5]. In the present study, only one patient among all patients was followed up as intubated. The patient died after 17 days of intensive care hospitalization.

As a treatment protocol, 1000 IU of the C1 inhibitor concentrate was administered to 68.6% of the patients, and the median administration duration value was 30 minutes in the ED. Of the patients, 11.4% were hospitalized, and the median length of hospital stay was 4 days (range: 1–17 days). All the hospitalized patients had recurrent emergency service admissions in the last 6 months, they all had type 1 HA, and all had a family history. Also, the localization of the attack was the GIS in 1 patient and facial swelling accompanied by additional laryngeal edema in 3 patients. No statistically significant association was found between angioedema localizations and the decision to hospitalize and discharge (*p* > 0.05). Four patients were admitted to the hospital 1 day after the onset of symptoms. 1000 IU of the C1 inhibitor concentrate was administered to all 4 patients who were hospitalized, and FFP treatment was given to 1 patient in addition to C1 inhibitor concentrate treatment. Although all of them had a previous diagnosis, their symptoms did not regress with the treatment given in the ED, and there were no complete recoveries. However, the mean duration to administer the C1 inhibitor concentrate to these patients was 142.5 minutes.

The long duration of the onset of attack treatment in the ED is important for the decision of hospitalization/discharge.

The LACE index is developed to predict admissions to the ED in the last 6 months [13]. In the present study, it was observed that those who were admitted to the hospital in the last 30 days had a higher rate of admission to the hospital in the last 6 months (*p* < 0.001). The LACE index at admission predicted 30 days of admission with (AUC = 0.75, 95% CI (0.56–0.91)) acceptable discrimination. In the present study, the LACE index of patients with recurrent admissions in the last 6 months was calculated as 4.2 and the LACE index of patients with a single admission was calculated as 4.7. In statistics, the LACE index was found to be significant in 30-day readmissions (*p* = 0.030).

None of the angioedema attack localizations were associated with the LACE risk (*p* > 0.05). In the patient group, the face and uvula were seen together in 5 people as the attack localization, whereas it was the lips in 1 person in addition to these locations. The combined localization of the abdomen and trunk was observed in 1 patient. Also, multiple localizations were not associated with the LACE risk and 30-day emergency admission (*p* > 0.05).

## 5. Limitations

The results of the present research should be assessed in light of some limitations. First, it was a single-center, retrospective study, and hence, the sample size was limited. Therefore, multicenter studies with larger samples are needed to prospectively validate the scoring systems. Second, it was difficult to diagnose HA in the ED. The lack of genetic analyses to confirm the diagnosis was a limitation of the present research; therefore, such a study focusing on the mentioned subject is suggested.

## 6. Conclusion

Considering the number of patients admitted to the ED in a total of 12 years, 35 patients may seem relatively low. However, the average duration of diagnosis of HA patients has been reported to be 20 years [9]. Despite the availability of current guidelines for specific treatments in the treatment of HA attacks, HA morbidity and mortality rates remain important.

The LACE index and the number of admissions in the last 6 months included in the evaluation can be considered predictive in recurrent emergency service admissions of HA patients. However, the distribution of LACE-risk groups is not a priority. Therefore, the low-, medium- or high-risk level of the LACE index values should not be taken into account in the readmission of these patients.

Despite the treatment given in the ED, the persistence of the symptoms of patients affects the decision of hospitalization. The longer the duration of HA attack treatment, the higher the probability of hospitalization. As soon as HA patients apply to the ED, their attacks should be recognized, and treatment should be initiated accordingly in the shortest time possible.

## Figures and Tables

**Table 1:** Descriptive characteristics of patients.

Sex (*n*/%)		
Female	19	54.3
Male	16	45.7
The number of admissions in the last 6 months (mean/SD)	1.4	0.7
Age at admission (years) (mean/SD)	36.4	13
Systolic blood pressure (mmHg) (mean/SD)	128.1	22.3
Pulse (per minute) (mean/SD)	91	21.3
Temperature (°C) (mean/SD)	36.4	0.4
Respiratory rate (per minute) (mean/SD)	19.6	1.4
Saturation O_2_ (mean/SD)	97	1.8
AVPU (*n*/%)		
A: alert	34	97.1
U-intubated	1	2.9
Oxygen need (n/%)		
Absent	34	97.1
Present	1	2.9
Abdominal pain (*n*/%)		
Absent	22	62.9
Present	13	37.1
Chest pain (*n*/%)		
Absent	34	97.1
Present	1	2.9
Nausea and vomiting (*n*/%)		
Absent	32	91.4
Present	3	8.6
Facial swelling		
Absent	24	68.6
Present	11	31.4
Body swelling (*n*/%)		
Absent	26	74.3
Present	9	25.7
Sore throat (*n*/%)		
Absent	26	74.3
Present	9	25.7
Hoarseness (*n*/%)		
Absent	32	91.4
Present	3	8.6
Shortness of breath (*n*/%)		
Absent	28	80
Present	7	20
Headache (*n*/%)		
Absent	35	100
Present	0	0
Syncope/near syncope (*n*/%)		
Absent	33	94.3
Present	2	5.7
Difficulty swallowing (*n*/%)		
Absent	34	97.1
Present	1	2.9
Urticaria (*n*/%)		
Absent	35	100
Present	0	0
Localization of HA attack^*∗*^(*n*/%)		
Abdomen	13	37.2
Uvula	15	42.9
Face	10	28.6
Lips	2	5.8
Trunk	3	8.6
Duration of swelling (hours) (mean/SD)	25.2	32.4
Family history (*n*/%)		
Absent	3	8.6
Present	32	91.4
HA type (*n*/%)		
Acquired	1	2.9
Type 1	28	80
Type 2	6	17.1
Trauma relation (*n*/%)		
Absent	34	97.1
Present	1	2.9
Comorbidity (*n*/%)		
Absent	28	80
Present	7	20
Treatment adopted (*n*/%)		
C1 inhibitor concentration	24	68.6
Symptomatic	7	20
Fresh frozen plasma	2	5.7
Fresh frozen plasma + C1 inhibitor concentration	2	5.7
Hospitalization (*n*/%)		
Discharge	31	88.6
Hospitalization	4	11.4
C1 inhibitor concentrate application time (min) (median/SD)	63	60.3
Hospital stay (days) (mean/SD)	6.5	7.5
CCI (mean/SD)	0.3	0.8
NEWS (mean/SD)	1.7	2
NEWS2 (mean/SD)	1.7	2
LACE index (mean/SD)	4.3	1.9
LACE risk (*n*/%)		
Low	25	71.4
Moderate	8	22.9
High	2	5.7

^*∗*^Some patients had more than one localization.

**Table 2:** Key characteristics of the study population by readmission within 30 days.

	The number of hospital admissions						*p* value
	0 (*n* = 28)		1 (*n* = 7)		Total (*n* = 35)		
The number of admissions in the last 6 months (median/range)	1	1–3	2	2-3	1	1–3	**<0.001** ^*∗*^
LACE index (median/range)	4	3–11	5	4–6	4	3–11	**0.030** ^*∗*^
LACE risk (*n*/%)							
Low	22	78.6	3	42.9	25	71.4	0.069^†^
Moderate	4	14.3	4	57.1	8	22.9	
High	2	7.1	0	0	2	5.7	

^*∗*^Wilcoxon rank-sum test. ^†^Fisher's exact test for count data. Each subscript letter denotes a subset of the number of hospital admission categories whose column proportions do not differ significantly from each other at the 0.05 level. Statistically significant values are expressed in bold.

## Data Availability

The datasets used and/or analyzed during the current study are available from the corresponding author on reasonable request.
